# Cancer Hacks Neurodegeneration: Cystatin-C as a Bridge for Microglial Amyloid Clearance via TREM2

**DOI:** 10.34133/research.1456

**Published:** 2026-10-14

**Authors:** Yuanhao Zhang, Conglei Li, Chenzhong Li

**Affiliations:** ^1^ Institute of Precision and Regenerative Medicine, Department of Biomedical Sciences, Division of Biomedical Health Sciences, School of Medicine, The Chinese University of Hong Kong, Shenzhen, Guangdong, China.; ^2^ Division of Clinical Medicine and Health Network, School of Medicine, The Chinese University of Hong Kong, Shenzhen, Guangdong, China.; ^3^ Guangdong Basic Research Center of Excellence for Aggregate Science, School of Science and Engineering, The Chinese University of Hong Kong, Shenzhen, Guangdong, China.; ^4^ Department of Biomedical Engineering, Division of Biomedical Health Sciences, School of Medicine, The Chinese University of Hong Kong, Shenzhen, Guangdong, China.

## Abstract

Historically, epidemiological studies have consistently highlighted an inverse correlation between cancer and Alzheimer’s disease, yet the molecular link between these 2 distinct pathologies remained elusive. In a recent breakthrough published in *Cell*, Li et al. provide a compelling answer by revealing that peripheral tumors secrete cystatin-C, which binds to preexisting amyloid oligomers and activates triggering receptor expressed on myeloid cells 2 (TREM2) in microglia, leading to the clearance of preexisting amyloid plaques in Alzheimer’s disease mice.

Amyloid-beta (Aβ) peptides are central pathological hallmarks of Alzheimer’s disease (AD), originating from the sequential proteolytic cleavage of the amyloid precursor protein [1]. Aβ peptides exist in multiple assembly states, ranging from simple monomers to soluble oligomers [2]. Critically, soluble Aβ oligomers are now recognized as the most neurotoxic species [3]. Traditionally, research on cancer-derived secreted proteins (CSPs) focused on their roles in remodeling the tumor microenvironment and driving paraneoplastic syndromes [4,5]. Among CSPs, cystatin-C (Cyst-C) emerges as a particularly promising neuroprotective candidate, opening new avenues for AD by clearing Aβ oligomers.

In *Cell* 2026, Li et al. established multiple allograft tumor models in 5×FAD mice and found that the presence of peripheral tumors markedly reduced Aβ pathology. Building on the hypothesis that peripheral cancer exerts a systemic influence on central neurodegeneration, Li et al. proved that CSPs are effector molecules responsible for inhibiting amyloid pathology, identified Cyst-C as a key therapeutic mediator [6], and confirmed that peripherally administered human Cyst-C effectively crosses the blood–brain barrier (BBB) to reach the brain of 5×FAD mice. To elucidate the central mechanism, the authors then identified that it was triggering receptor expressed on myeloid cells 2 (TREM2), an immune receptor expressed on the surface of microglia associated with AD [7], that binds with Cyst-C. Upon binding, Cyst-C triggers the interaction between the obligate adapter DNAX activating protein of 12 kDa (DAP12) and TREM2. Collectively, these findings reveal a therapeutic cascade: tumor-derived Cyst-C as Aβ carrier → TREM2/DAP12 activation → microglial Aβ clearance (Fig. 1).

**Fig. 1.**
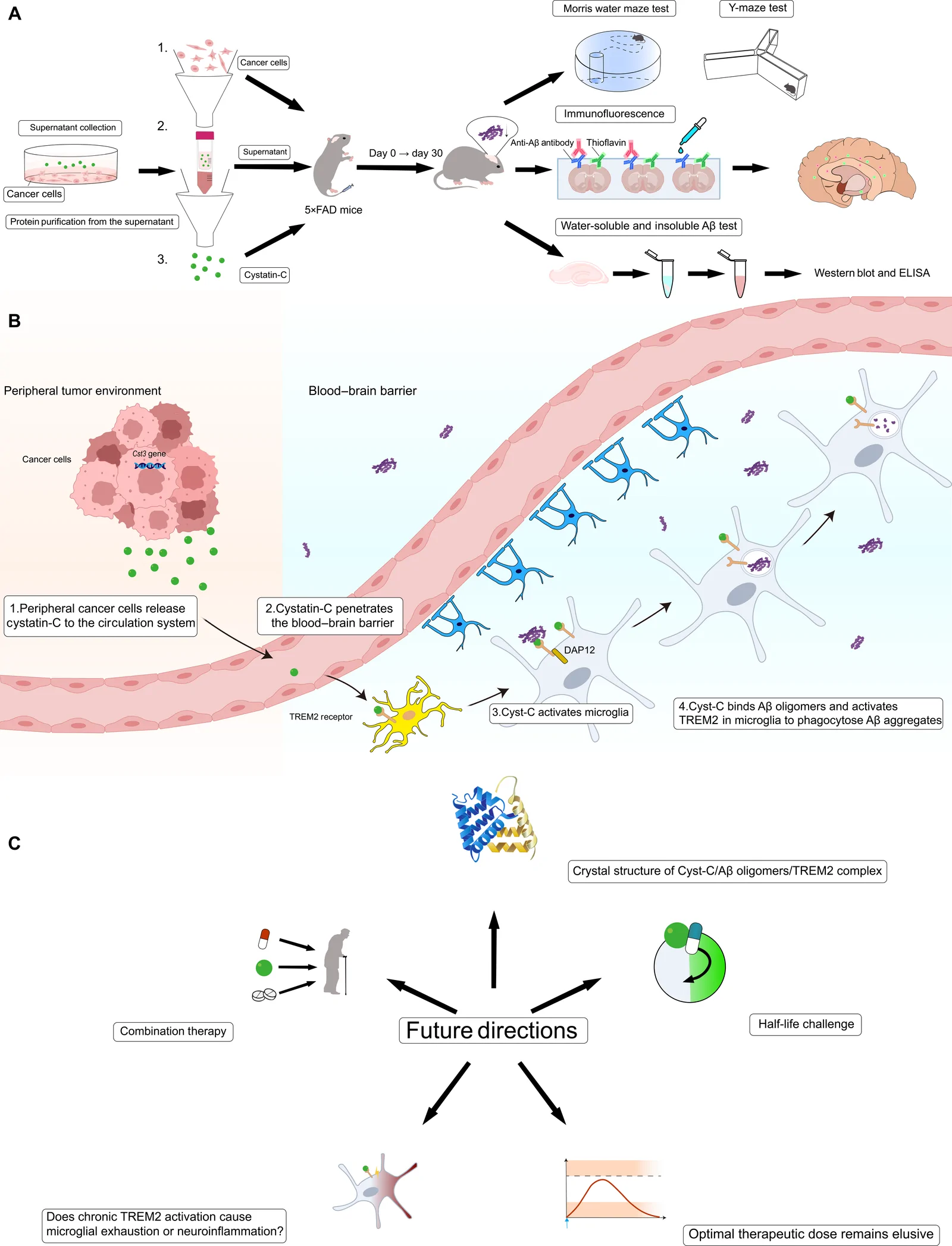
Cystatin-C acts as a bridge for microglial amyloid clearance via triggering receptor expressed on myeloid cells 2 (TREM2). (A) Li et al. demonstrated that peripheral tumor cells, their secreted proteins (cancer-derived secreted proteins [CSPs]), and purified cystatin-C (Cyst-C) each independently reduced amyloid plaque burden and ameliorated the pathogenesis of Alzheimer’s disease (AD), as assessed by cognitive tests, immunofluorescence, enzyme-linked immunosorbent assay (ELISA), and Western blot. (B) Cyst-C, encoded by *Cst3* and secreted by cancer cells, crosses the blood–brain barrier and activates the microglial clearance of amyloid-beta (Aβ). (C) The therapeutic potential of Cyst-C may be further enhanced through combination with existing immunotherapies, while future efforts to optimize dosing, extend half-life, and refine its structure will be critical for clinical translation.

These findings reveal a novel cross talk between oncology and neurobiology, where a cancer-secreted protein directly triggers microglial amyloid clearance. Clinically, this discovery introduces a therapeutic paradigm and differs from current anti-Aβ antibodies, clearing preexisting plaques via the Cyst-C–TREM2 axis. By leveraging this endogenous mechanism, it offers a more promising strategy for reversing established pathology in symptomatic AD patients, especially after recent failure of AL002 (anti-TREM2 monoclonal antibody) in a phase 2 trial [8].

The therapeutic mechanism of AL002 involves clustering TREM2 receptors on microglia to trigger downstream signaling while simultaneously inhibiting TREM2 shedding, thereby markedly reducing the formation of soluble TREM2 (sTREM2). Accumulating evidence shows that sTREM2 has its protective role against amyloid pathology by activating microglial phagocytosis as well as blocking Aβ aggregations [9,10]. Furthermore, as a biomarker, its increase in cerebrospinal fluid strongly correlates with a decelerated rate of cognitive decline [11]. However, in the INVOKE-2 phase II trial, despite sustained TREM2 activation achieved by AL002 in the central nervous system, it failed to translate into amyloid clearance and paradoxically accelerated brain atrophy [12], and the underlying mechanisms remain to be investigated.

In contrast, the endogenous Cyst-C–TREM2 axis offers a fundamentally distinct approach. Functioning as a multivalent complex builder with Aβ oligomers, Cyst-C may serve a dual role as both an Aβ carrier and a TREM2 agonist. Li et al. showed that Cyst-C (~13 kDa) penetrates the BBB of 5×FAD mice with an efficiency comparable to that of ^125^I-labeled dextran (~10 kDa), a bacterial glucose polymer that does not engage any known BBB transporter. This comparison suggests that Cyst-C crosses the pathologically compromised barrier largely via a receptor-independent, passive route—although the involvement of additional transport mechanisms cannot be fully excluded. Its low molecular weight also confers a clear penetration advantage over 150-kDa monoclonal antibodies such as AL002. Despite these advantages, the precise molecular mechanisms underlying the interactions of Cyst-C with TREM2 and sTREM2 require further investigation. Consistent with existing TREM2-targeting AD therapy, we hypothesized that Cyst-C, rather than through forcing bivalent extracellular clustering and shedding blockade as AL002 does, acts as an endogenous TREM2 ligand like apolipoprotein E (ApoE) or as a “molecular glue” stabilizing the TREM2–DAP12 complex—comparable to the small-molecule agonist VG-3927 [13]. This shedding-sparing mechanism, if validated, would enable robust TREM2 activation while preserving endogenous sTREM2, positioning Cyst-C as a mechanistically distinct therapeutic model for AD.

Moreover, given recent evidence that peripheral blood or bone-marrow-derived cells can contribute to the microglial pool under neurodegenerative conditions [14], it has yet to be determined whether Cyst-C also engages TREM2 on newly recruited myeloid cells, potentially broadening its phagocytic effects beyond resident microglia. Given AD’s multifactorial pathology, often compounded by neuroinflammation—combination therapeutic strategies are increasingly favored. It warrants future investigation whether Cyst-C is synergistic with existing immunomodulatory therapies, including programmed cell death protein 1 (PD-1) and granulocyte-macrophage colony-stimulating factor (GM-CSF)-based approaches [15,16].

Although this study identified that Cyst-C binds to both Aβ_1-42_ oligomers and TREM2, the crystal structure of this complex needs to be resolved (Fig. 1). Future studies will be needed to clarify whether different cancer types differentially modulate Aβ aggregation and whether different Aβ species possess tumor specificity. Furthermore, the intrinsic properties of Cyst-C merit further investigation. Recent data suggest that it might also modulate inflammation reaction through other immune receptors [17]. These activities raise particular concerns when considering the clinical context: unlike the immunocompetent mice used by Li et al., AD patients often present with immune dysfunction. The behavior of Cyst-C—particularly its immunogenicity upon repeated administrations—in such compromised immune settings remains to be investigated. Based on this hypothesis, the development of Cyst-C-derived mimetic peptides should be prioritized, analogous to the strategy used for ApoE (e.g., COG1410) [18], to retain the specific domain responsible for TREM2 activation while eliminating the regions associated with off-target effects.

Clinically, despite the finding by Li et al. that Cyst-C can penetrate the BBB in AD mice, its applicability to patients with a relatively intact BBB, particularly in early-stage disease, represents a key open question, considering the differences between murine and human BBB [19]. Although its low molecular weight (~13 kDa) acts as an advantage in penetrating BBB, it may also pose a half-life challenge due to rapid renal clearance. This is because for a healthy adult human kidney, exogenous proteins of a small size (approximately 10 kDa) will be generally filtered readily through the glomerulus, leading to shorter durations in blood circulation, although the charges of proteins also affect their renal clearance rate. However, conjugating Cyst-C to a macromolecular carrier could compromise its brain penetration. Alternative routes, such as receptor-mediated delivery via chaperone peptides, warrant future explorations [20]. Given the clinical heterogeneity of AD patients, the optimal therapeutic dose of Cyst-C and whether its TREM2 activation could provoke AL002-like side effects and overactivation remain unknown [8].

Overall, the study by Li et al. represents a conceptual advance in neuroimmunology, demonstrating that a cancer-secreted protein can engage TREM2 to promote microglial Aβ clearance. Building on prior evidence of its role in preventing amyloid aggregation, Cyst-C emerges as a promising therapeutic candidate for AD, with potential applications spanning the entire disease continuum from prophylaxis to treatment. Although substantial hurdles remain for clinical translation, this work opens a new therapeutic frontier for AD.
